# A Liposome-Based Mycobacterial Vaccine Induces Potent Adult and Neonatal Multifunctional T Cells through the Exquisite Targeting of Dendritic Cells

**DOI:** 10.1371/journal.pone.0005771

**Published:** 2009-06-03

**Authors:** Arun T. Kamath, Anne-Françoise Rochat, Dennis Christensen, Else Marie Agger, Peter Andersen, Paul-Henri Lambert, Claire-Anne Siegrist

**Affiliations:** 1 World Health Organization Collaborating Center for Vaccinology and Neonatal Immunology, Departments of Pathology-Immunology and Pediatrics, Medical Faculty of the University of Geneva, Geneva, Switzerland; 2 Department of Infectious Disease Immunology, Adjuvant Research, Statens Serum Institut, Copenhagen, Denmark; New York University School of Medicine, United States of America

## Abstract

**Background:**

In the search for more potent and safer tuberculosis vaccines, CAF01 was identified as a remarkable formulation. Based on cationic liposomes and including a synthetic mycobacterial glycolipid as TLR-independent immunomodulator, it induces strong and protective T helper-1 and T helper-17 adult murine responses to Ag85B-ESAT-6, a major mycobacterial fusion protein. Here, we assessed whether these properties extend to early life and how CAF01 mediates its adjuvant properties *in vivo*.

**Methods/Findings:**

Following adult or neonatal murine immunization, Ag85B-ESAT-6/CAF01 similarly reduced the post-challenge bacterial growth of *M. bovis* BCG, whereas no protection was observed using Alum as control. This protection was mediated by the induction of similarly strong Th1 and Th17 responses in both age groups. Multifunctional Th1 cells were already elicited after a single vaccine dose and persisted at high levels for at least 6 months even after neonatal priming. Unexpectedly, this potent adjuvanticity was not mediated by a massive targeting/activation of dendritic cells: in contrast, very few DCs in the draining lymph nodes were bearing the labeled antigen/adjuvant. The increased expression of the CD40 and CD86 activation markers was restricted to the minute portion of adjuvant-bearing DCs. However, vaccine-associated activated DCs were recovered several days after immunization.

**Conclusion:**

The potent adult and neonatal adjuvanticity of CAF01 is associated *in vivo* with an exquisite but prolonged DC uptake and activation, fulfilling the preclinical requirements for novel tuberculosis vaccines to be used in early life.

## Introduction

Considerable efforts are aiming at the development of novel, safer and more efficacious vaccines against tuberculosis [1]–[4]. Despite the lack of reliable biomarkers of protective immunity against *M. tuberculosis* (Mtb), it is currently considered that these vaccines should elicit anti-mycobacterial CD4^+^ effector T cells producing type 1 cytokines, as highlighted by the severity of mycobacterial infections when interleukin (IL)-12/interferon (IFN)-γ [5], [6] or tumor necrosis factor (TNF)-α [7] responses are impaired. An important protective role for T-helper 17 (Th17) cells is also suspected in the recruitment of Th1 cells to the lung [8], [9].

Exposure to Mtb may occur very early in life and infections with Mtb are frequently severe in infants and young children whose immature immune system fails to limit bacterial spread [10]. A cornerstone of the global tuberculosis control program is thus to immunize with Bacillus Calmette Guerin (BCG) soon after birth in areas of high tuberculosis incidence. BCG vaccination is currently quite effective (approximately 80%) in protecting human infants from disseminated forms of disease including meningitis and miliary TB [11]. It induces adult-like IFN-γ responses [12], [13], probably as a result of prolonged replication and potent dendritic cells (DC) activation and BCG-induced T cells express a vast array of cytokine and phenotypic profiles [14], [15]. However, BCG has been unreliable as a vaccine to prevent the pulmonary form of TB. In addition, it may lead to severe disseminated BCGitis in HIV-1 infected children such that WHO now officially recommends that HIV-1 infected infants not to be immunized with BCG [16]. As areas of high tuberculosis and HIV-1 prevalence partly overlap, novel tuberculosis vaccines should induce potent and sustained anti-mycobacterial responses early in life, while preferably using non-replicating vaccines to avoid safety issues in neonates and immunodeficient patients. Unfortunately, the Th1 response capacity of human neonates is limited (reviewed in [17]) and whether adult levels of Th17 effector cells may be elicited in early human life is yet unknown. Thus, whether novel subunit tuberculosis vaccine candidates will eventually prove effective in infants is difficult to predict.

Although there is ample evidence that mice may not be reliably used to predict human vaccine efficacy [18], the main stages of immune maturation are sufficiently well conserved between humans and mice for specific neonatal animal models to reproduce infant B and T cell response patterns [19]–[22]. We and others have reported that the limitations of neonatal T cell responses can be overcome by some specific vaccines and/or through potent DC activation signals [12], [23], [24]. However, aluminium salts, the only adjuvants currently licensed for use in infants, exacerbate the Th2-like profile of neonatal responses [25], [26]. We recently reported that the novel IC31® adjuvant elicits adult-like multifunctional neonatal CD4^+^ T cells against a fusion protein of two major tuberculosis antigens (Ag85B and ESAT-6) [27]. IC31® contains a KLK peptide and a non-CpG oligonucleotide mediating DC activation in a toll-like receptor (TLR)-9 dependent manner [28], [29]. However, human neonatal DC respond poorly to TLR-9 signals [30], [31] and whether IC31® will be effective in early life is yet unknown.

Efforts to develop novel formulations have identified CAF01 (previously referred to as DDA-TDB or Lipovac) as another promising adjuvant. CAF01 is based on cationic liposomes formed by quaternary ammonium lipid N,N′-dimethyl-N,N′-dioctadecylammonium (DDA) incorporating the synthetic mycobacterial immunomodulator α,α′-trehalose 6,6′-dibeheneate (TDB) [32]–[34]. Compared to a panel of commercially available adjuvants, CAF01 was particularly effective in generating strong Th-1 and Th-17 T cell responses as well as strong antibody responses [33]–[35]. Strong immunogenicity and protective efficacy of Ag85B-ESAT-6 and CAF01, which will soon enter into a Phase I clinical trial, was demonstrated in mice, guinea pigs and monkeys [34], [36]–[38]. DDA has been reported to increase antigen uptake and presentation *in vitro* by bone-marrow derived DC [39], and TDB elicits a potent activation of innate immunity including macrophages and DCs. Remarkably, this TDB-induced activation is TLR-independent and mediated by the Syk-Card9-Bcl10-Malt1signaling pathway [35]. One hypothesis was that the strong potency of CAF01 was mediated by a massive targeting/activation of DCs. Ag85B-ESAT-6/CAF01 thus provided a unique opportunity to test this hypothesis and to assess the adjuvanticity of cationic based liposomes and the function of the Syk-Card9-NFkB signalization pathway in early life.

## Results

### Protective efficacy of neonatal immunization against mycobacterial infection

**Figure 1:**
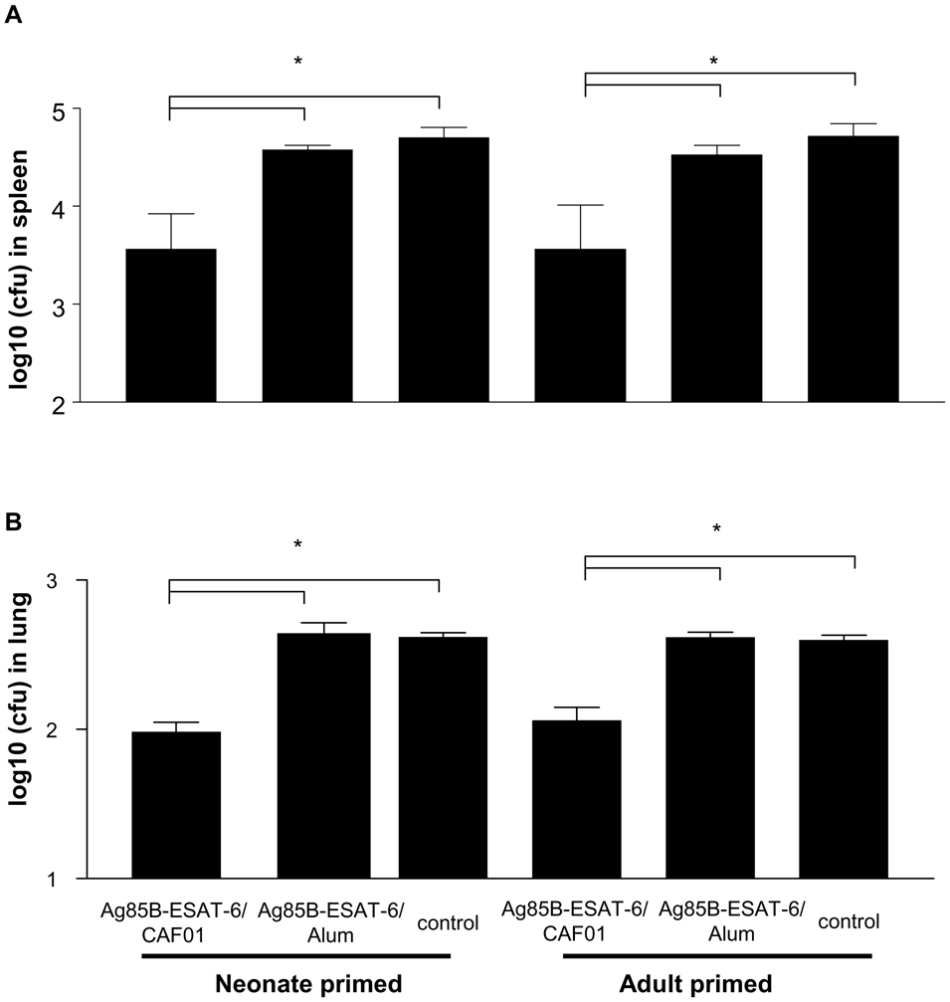
Ag85B-ESAT-6/CAF01 protects neonates and adults from mycobacterial infection. Immunized neonatal and adult mice were challenged with *Mycobacterium bovis* BCG i.v. The CFU in spleen (A) and lung (B) (mean and SD, 6–8/group) were determined four weeks later, and is representative of 2 independent experiments. *, p<0.05, differences between CAF01 and Alum or control were significant.

C57BL/6 mice were primed at 1 week of age (i.e. at the stage of immune maturation that most closely reflect that of human neonates [19]) or as adult controls with Ag85B-ESAT-6 (5 µg) formulated in CAF01 or Alum (aluminum hydroxide: negative control). Neonatal weight gain, a sensitive method of monitoring neonatal reactogenicity, was normal in each group and local reactions were not detected (data not shown). Mice were boosted 3 weeks later and challenged i.v. with *Mycobacterium bovis* BCG six weeks after boosting, to investigate whether neonatal and adult immunization would confer similar or distinct protection against a model mycobacterial infection. Ag85B-ESAT-6/Alum did not confer any protective efficacy, the number of CFU in spleen (Fig. 1A) and lungs (Fig. 1B) being as high as in control mice. In contrast, significantly lower bacterial counts were recovered from mice immunized with Ag85B-ESAT-6 in CAF01 either as adults or as neonates (Figure 1). Injection of CAF01 alone does afford any protection against mycobacterial infection. [34]. Thus, Ag85B-ESAT-6/CAF01 was selected for the further evaluation of its immunogenicity and DC targeting/activation capacities.

### Induction of adult-like multifunctional neonatal CD4^+^ Th1 cells

**Figure 2:**
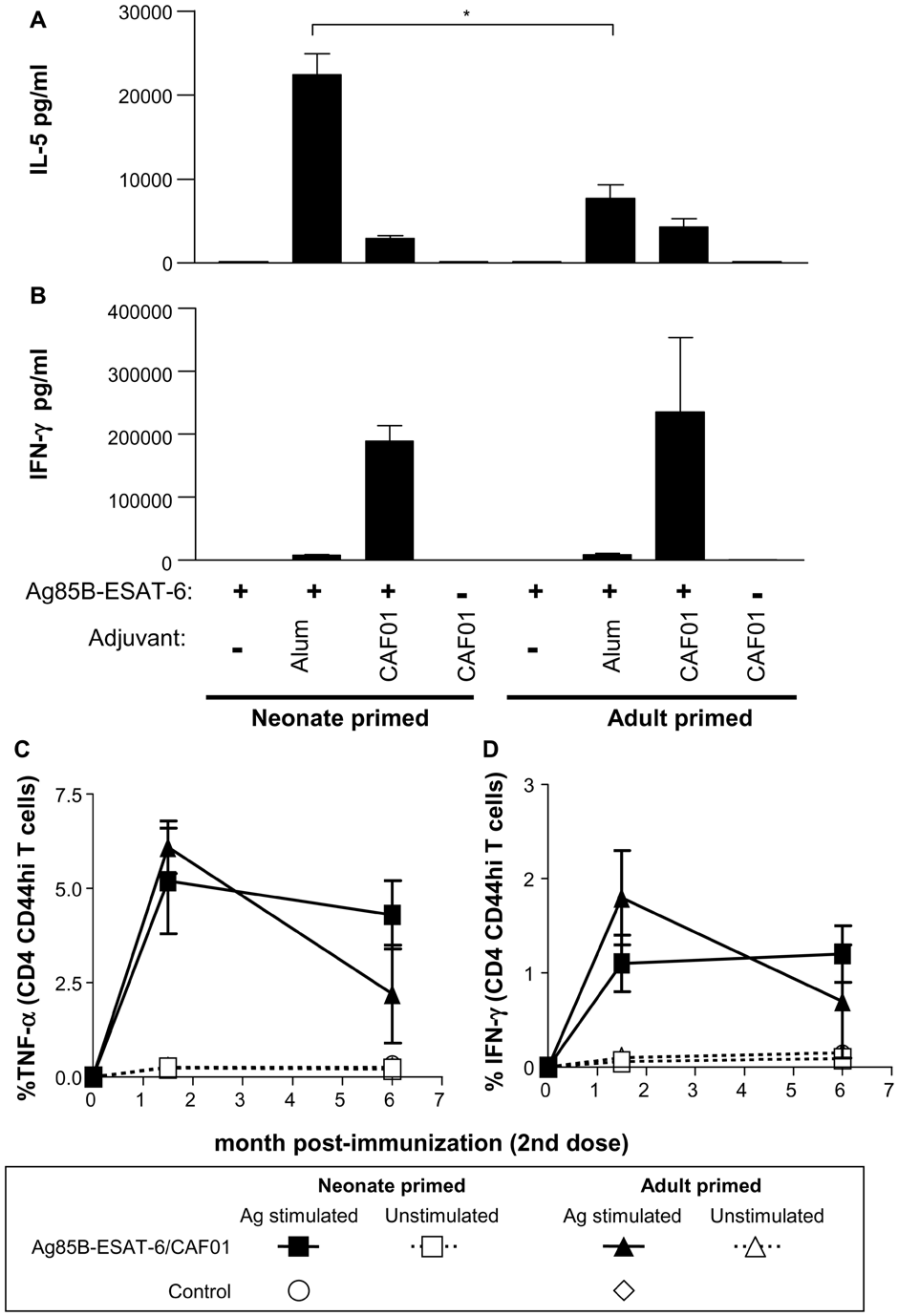
Ag85B-ESAT-6/CAF01 induces sustained adult-like Th1 cell responses in neonates. (A–B). Three weeks after the second immunization, splenocytes from neonatal and adult immunized mice were restimulated with antigen for 3 days. The production of IL-5 (A) and IFN-γ (B) was measured by ELISA. Cytokine production by unstimulated splenocytes was below the limit of detection. (C–D). Six weeks or 6 months after boosting, Ag85B-ESAT-6 specific IFN-γ (C) and TNF-α (D) producing cells were quantified by ICS. *, p<0.05 signifies differences between neonates and adult mice. Data is represented by the mean and SD of groups of at least 6 individual mice, and is representative of 2–3 independent experiments.

Following neonatal immunization, Ag85B-ESAT-6/Alum elicits weaker IFN-γ and stronger IL-5 responses in neonates than in adults, a Th2-preferential pattern characteristic of neonatal vaccine responses (Fig. 2A and [27]). In contrast, a mirror pattern consisting of similarly high IFN-γ and modest IL-5 responses was induced by Ag85B-ESAT-6/CAF01 in adults and neonates. The induction of adult-like neonatal responses was confirmed by a similar frequency of IFN-γ and TNF-α producing cells in both age groups, both early (6 weeks) and late (6 months) after boosting (Fig. 2 B). These similarities extend to IL-2 and also to IL-17-producing cells (Table 1 and data not shown). Cytokines (IFN-γ, TNF-α, IL2 and IL-17) producing cells were not detected in splenocytes from H1/CAF01 immunized mice restimulated with medium only, nor in stimulated and unstimulated splenocytes from naïve mice or mice injected with only H1 or CAF01 (Fig. 2 and data not shown). The adult-like responses did not result from the postnatal maturation of the immune system, as adult-like Th1 responses were already elicited after a single neonatal Ag85B-ESAT-6/CAF01 immunization (Table 1). In accordance with results in adults [33], Ag-specific CD8^+^ T cells were not detected, indicating that the protective efficacy of Ag85B-ESAT-6/CAF01 is associated with the induction of Th1 and Th17 CD4^+^ T cells.

**Table 1:** Ag85B-ESAT-6-specific T cell responses following neonatal and adult immunization with Ag85B-ESAT-6 in the CAF01 adjuvant.

	In vitro stimulation^a^				Ex vivo intracellular cytokine staining^b^ (% of CD44^+^ CD4^+^ T cells)		
	Neonates	Adult		p value^c^	Neonates	Adult	p value
**2 doses**							
IFN-γ	738±57	789±255	ISC/10^6^ splenocytes	NS	1.1±0.3	1.8±0.5	NS
IL-2	9.6±1.5	12.8±2.4	U/ml	NS	4.6±1.3	5.3±0.7	NS
TNF-α	0.9±0.1	0.9±0.2	ng/ml	NS	5.2±1.4	6.1±0.7	NS
IL-17	30.5±3.5	22.4±2.4	ng/ml	NS	ND^d^	ND	
**1 dose**							
IFN-γ	13.5±1.6	10.4±3.2	ng/ml	NS	ND	ND	
IL-5	0.5±0.2	0.6±0.2	ng/ml	NS	ND	ND	

a IFN-γ secreting cells (ISC)/10^6^ splenocytes were determined after 48 hr of culture with antigen and IL-2 (bioassay), TNF-α, IL-17, IFN-γ and IL-5 (ELISA) after 72 hr of culture with antigen. Cytokine production by unstimulated splenocytes from mice immunized with Ag85B-ESAT-6/CAF01 and antigen stimulated/unstimulated splenocytes from mice immunized with Ag85B-ESAT-6 alone or CAF01 alone was below the limit of detection.

b Following 6 hr culture with antigen and co-stimulation (CD28/CD49d), percent of cytokine-producing cells determined by flow cytometry. Unstimulated splenocytes from mice immunized with Ag85B-ESAT-6/CAF01 and antigen stimulated/unstimulated splenocytes from unimmunised mice were <0.2%.

c NS: p>0.05.

d ND: not done.

**Figure 3:**
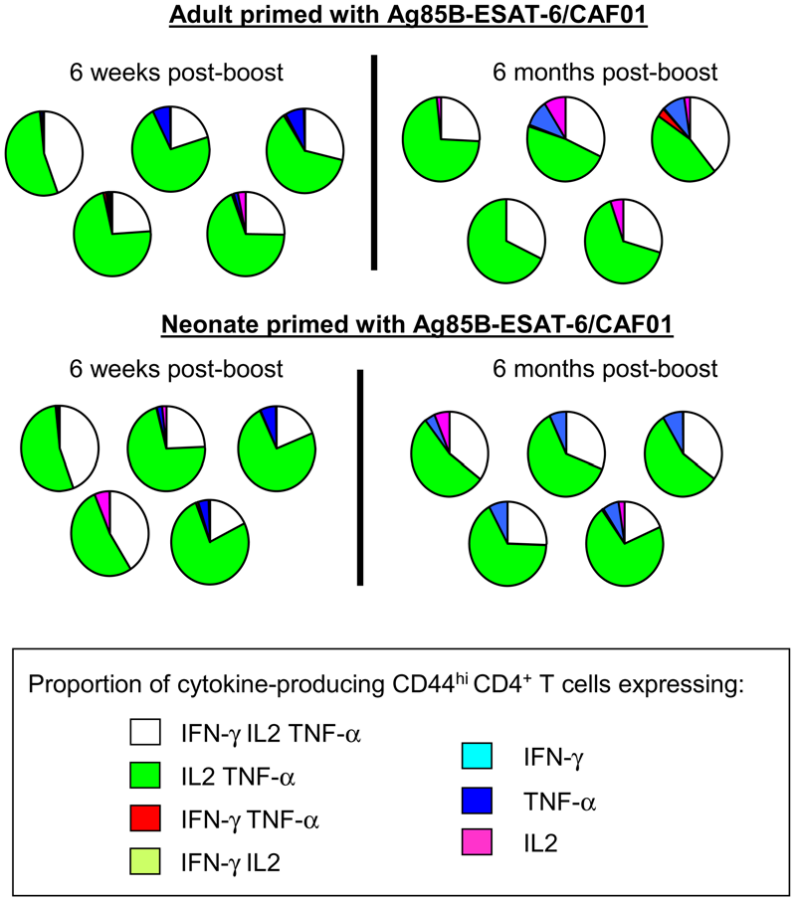
Ag85B-ESAT-6/CAF01 stimulates production of multifunctional CD4^+^ T cells in neonates and adults. The expression of IFN-γ, IL-2 and TNF-α was determined by ICS on splenocytes from neonatal and adult mice, 6 weeks and 6 months post-boost. Cytokine production was only detected in CD4^+^ T cells from Ag/CAF01-immunized mice stimulated with antigen. The concurrent expression of IFN-γ, IL-2 and TNF-α is represented as pie graphs of cytokine expression by CD4^+^ CD44^+^ T cells of individual mice, and is representative of 2 independent experiments.

To further delineate the functionality of Ag85B-ESAT-6 specific CD4^+^ T cells elicited in early life, their co-production of IFN-γ, TNF-α and IL-2 was assessed by flow cytometry. Six weeks after boosting, IFN-γ, TNF-α and IL-2 were produced by CD44^hi^ activated/memory CD4^+^ T cells (not shown). Combination gating to determine the cytokine production of single T cells indicated a similar pattern whether mice were immunized with Ag85B-ESAT-6/CAF01 in adult or early life (Fig. 3). Most cytokine^+^ T cells produced TNF-α/IL-2, often with IFN-γ. Remarkably, this multicytokine pattern was similar early (6 weeks) and late (6 months) after immunization. Thus, neonatal immunization with Ag85B-ESAT-6 in CAF01 elicits adult-like multifunctional CD4^+^ Th1-associated and Th17 responses that are significantly different from the Th2-biased neonatal responses induced with Alum.

### *In vivo* targeting and activation of neonatal and adult dendritic cells

**Figure 4:**
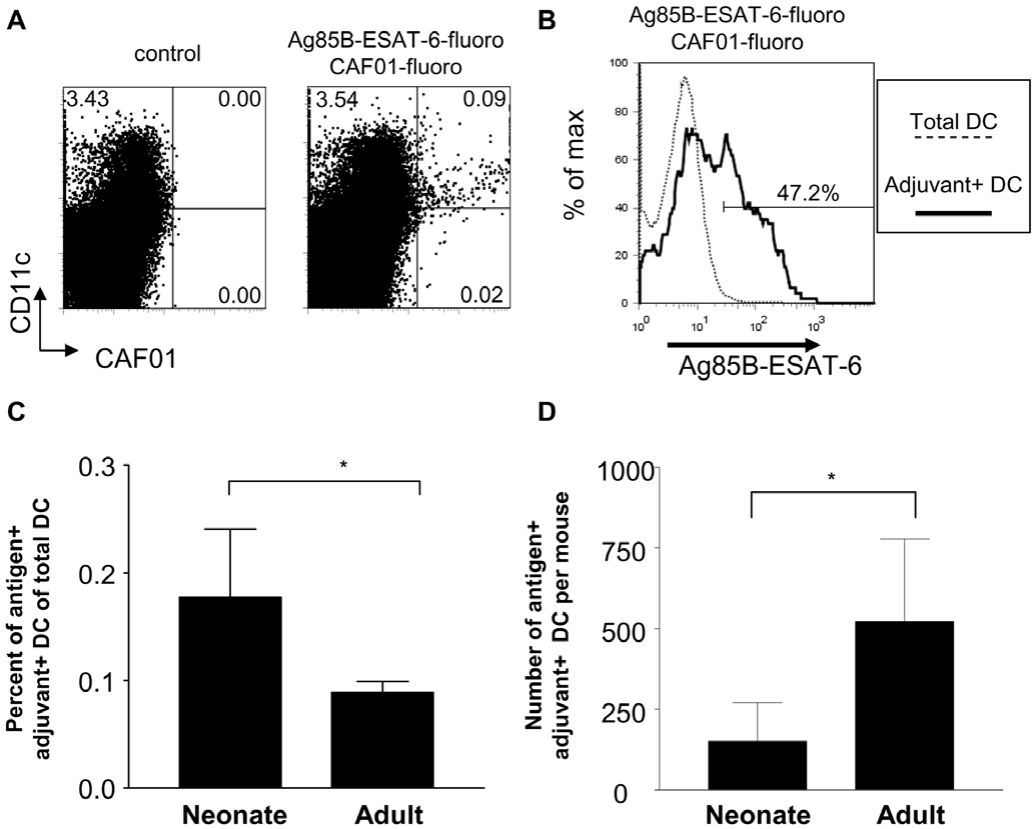
Targeting of Ag85B-ESAT-6 and CAF01 in neonatal and adult DC of the draining LN. Twenty-four hours after immunization, (A) the percentage of CAF01-NBD^+^ CD11c^+^ cells compared to all cells bearing the adjuvant in the draining LN was determined. In a representative dot plot of an adult mouse, the percent of cells in quadrants is shown. (B) The percent of Ag85-ESAT-6^+^ CAF01^+^ DC amongst all CAF01^+^ DC was determined. In a representative histogram from an adult mouse, the presence of Ag85-ESAT-6-FITC in CAF01^+^ DC (thick line) was calculated using total DC as the control histogram (dotted line). The percent (C) and number (D) of Ag85-ESAT-6^+^ CAF01^+^ DC was calculated. The data are expressed as mean and SD of groups of at least 4 individual mice, and is representative of 4 independent experiments. *, p<0.05 signifies differences between neonatal and adult mice.

We next asked whether the remarkably potent induction of adult-like Th1 and Th17 neonatal responses by Ag85B-ESAT-6/CAF01 resulted from the *in vivo* targeting and activation of a large number of DCs. The antigen and adjuvant were separately labeled with fluorochromes to track antigen (Ag^+^) and/or adjuvant (Adj^+^) DC in the draining lymph nodes (LN) after injection of Ag85B-ESAT-6 in CAF01 (Fig. 4A and B), as similarly preformed in [40]. Twenty-four hours after adult immunization, cells were detected in the draining LN associated with the fluorochrome-labeled formulation. Adj^+^ LN cells were mostly (84.2±4.8%) in the CD11c^+^ DC population, (Fig. 4A). Two populations were detected: Ag^+^Adj^+^ (52.6±14.0% of the total Adj+ DC) and Ag^−^Adj^+^ cells (Fig. 4B). These Adj^+^ DC were CD205^+^ CD8^−^, the phenotype of periphery-derived DC [41] (data not show). Both the proportion of total DCs (0.09±0.04%) and the number (523±254) of DCs per mouse that were Ag^+^Adj^+^ were low (Fig. 4C and D), indicating that CAF01 adjuvanticity was not mediated through an extensive DC targeting. A similar pattern was observed in neonates. Following a single immunization, Adj^+^ LN cells were also mostly (71.2±9.8%) observed in the CD11c^+^ DC population. Among Adj^+^ DCs, fewer cells were Ag^+^ and Adj^+^ DCs (28.6±8.6% vs 52.6±14.0%) in neonates than in adults. Despite a higher proportion of Ag^+^Adj^+^ DCs in the total neonatal DC population (0.18±0.06%), this translated into even lower numbers (151±119) of Ag^+^Adj^+^ DCs per 1-week-old mouse (Fig. 4) as a result of differences in organ cellularity.

**Figure 5:**
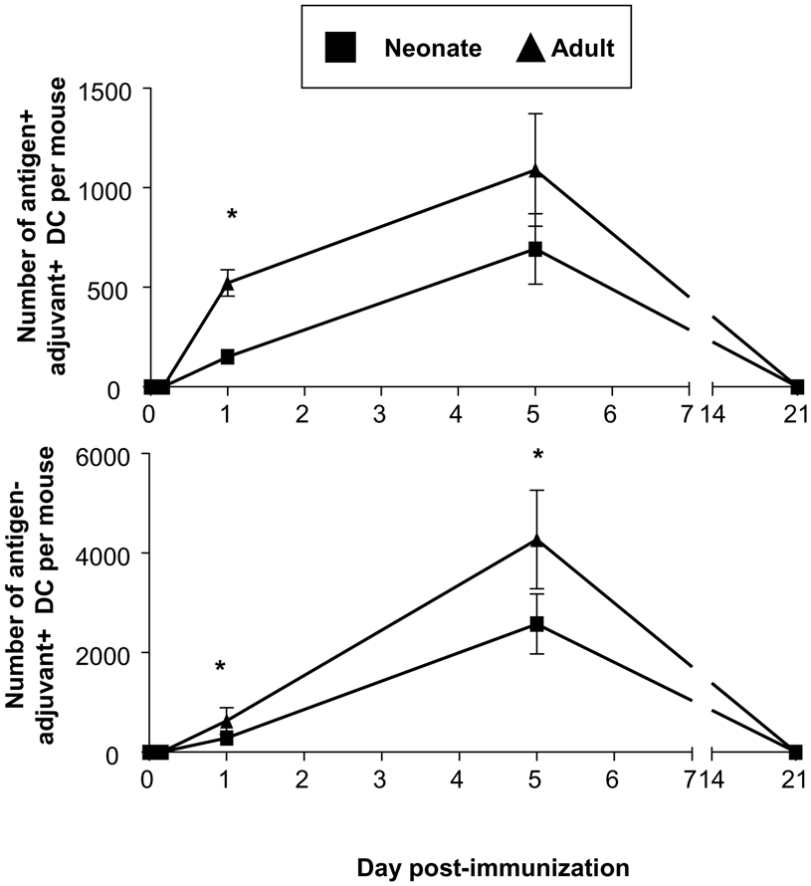
Kinetics of Ag85B-ESAT-6 and CAF01 labeled DC in the draining LN of adults and neonates. At various time points after immunization, the number of Ag85-ESAT-6^+^ CAF01^+^ DC and Ag85-ESAT-6^−^ CAF01^+^ DC was calculated. The data are expressed as mean and SD of groups of at least 4 individual mice. *, p<0.05 signifies differences between neonates and adult mice.

We postulated that such an exquisite DC targeting pattern could only be effective if sustained over time and studied the kinetics of Ag^+^Adj^+^ and Ag^−^Adj^+^ DCs in the draining LN at various time points following Ag85B-ESAT-6/CAF01 immunization. Neither Ag^+^ nor Adj^+^ DCs were identified 4 hours p.i., indicating that the formulation is retained efficiently at the injection site (Fig. 5). In adults, vaccine-labeled cells were first observed on Day 1, increased until Day 5 after immunization and were not visualized any more on Day 21. The migration kinetics were similar for Ag^+^Adj^+^ and Ag^−^Adj^+^ DCs and in both age groups (Fig. 5). Remarkably, there were very few vaccine-associated DCs (100–1000 Ag^+^Adj^+^ DCs and 200–5000 Ag^−^Adj^+^ DCs) at any time point assessed (Fig. 5).

**Figure 6:**
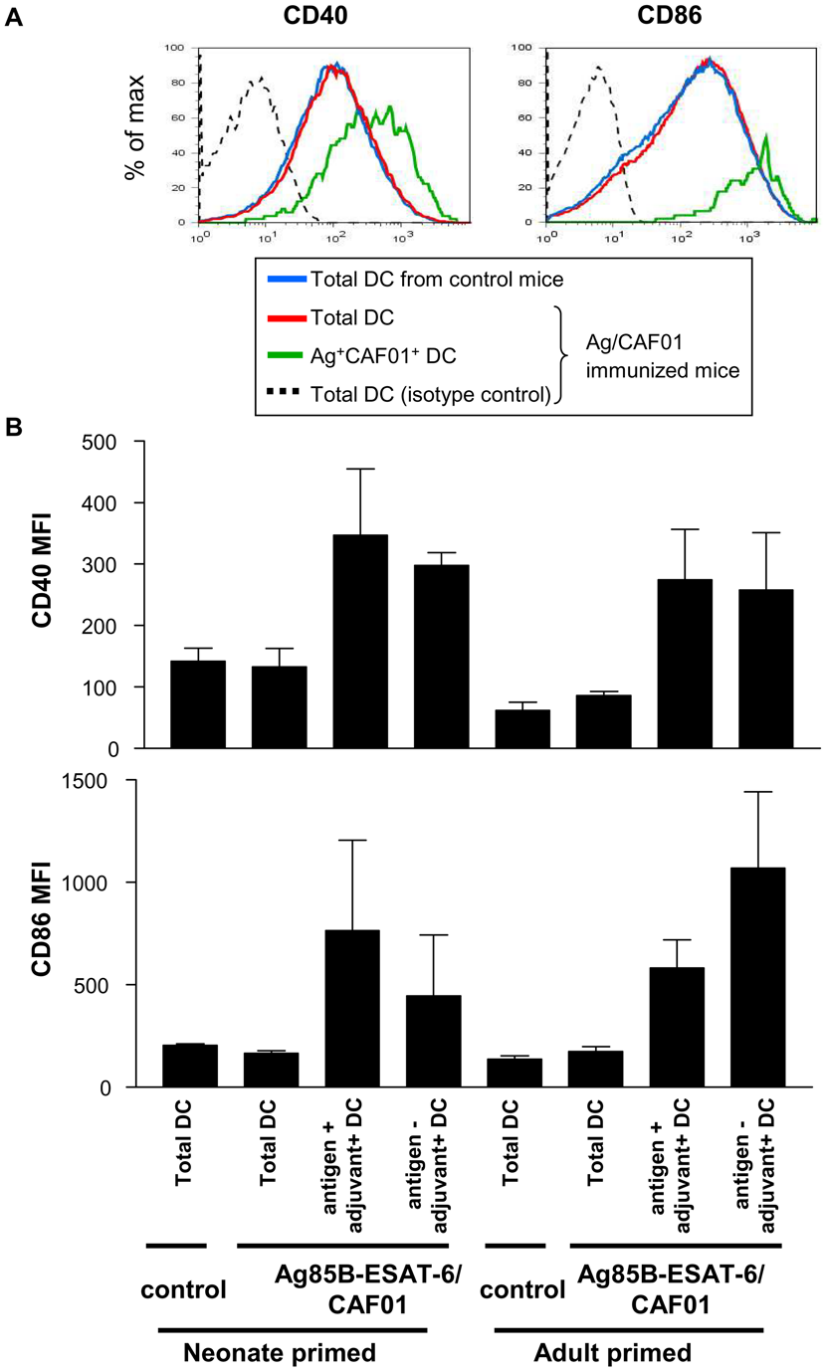
Targeted functional activation of DC in neonates and adults. Twenty-four hours after immunization, the expression of CD40 and CD86 by total DC and adjuvant^+^ DC was assessed. (A) Histograms gated on CD11c^+^ cells from adult mice immunized with Ag85B-ESAT-6/CAF01 represent the expression of co-stimulation molecules by total DC and CAF01^+^ DC. (B) The MFI is expressed as mean and SD of groups of at least 4 individual mice, and is representative of 4 independent experiments.

The apparent discrepancy between the induction of strong Th1/Th17 responses and the paucity of Ag^+^Adj^+^ DCs in the draining LN prompted us to define the activation pattern elicited by CAF01. Assessing the surface expression of the CD40 and CD86 co-stimulation molecules on all LN DCs indicated that CAF01 only activated Adj^+^ DCs (Fig. 6), without detectable extension to bystander DCs. DC activation was phenotypically similar in neonates and in adults. A classical DC activation marker of Th1-triggering responses, IL-12p40 expression, was not detected in any sample (not shown). Thus, the strong immunogenicity of Ag85B-ESAT-6/CAF01 in both adults and neonates is not supported by a massive DC targeting and activation pattern but in contrast by an exquisitely focused *in vivo* DC activation pattern sustained during at least several days.

## Discussion

Specific requirements have to be met for subunit vaccines to elicit potent Th1 CD4^+^ T cell responses, and these are even more stringent in early life given the preferential induction of Th2-biased CD4^+^ T cell neonatal responses by conventional vaccines [17], [42]. We show here that the novel adjuvant formulation CAF01 meets these requirements through the sustained targeting of a very small number of DCs and their activation through an IL-12 independent pathway.

The strong protective efficacy of Ag85B-ESAT-6/CAF01 against mycobacterial challenge [36]–[38], [43] and its capacity to induce potent Th1 and Th17 responses suggested that CAF01 mediated its adjuvanticity through a most efficient targeting and activation of dendritic cells, the key regulators of T- and B-cell immunity. Indeed, numerous vaccination strategies have shown that enhancing DC targeting *in vivo* was required for potent CD4^+^ and/or CD8^+^ T cell responses [44]. However, protective multifunctional Th1 and Th17 cells were elicited after 1 or 2 administrations of a formulation found to only label a minute DC population. Indeed, the number of vaccine-associated cells in the draining lymph nodes remained low (a few hundred DCs). We cannot formally exclude that the fluorochrome intensity of the labeled antigen and adjuvant in the *in vivo* setting may not reveal all the labeled cell population. However, the hypothesis that a successful vaccine requires general immune activation is not supported by the fact that upregulation of co-stimulation molecules is restricted to Adj^+^ DCs (Fig. 6 and data not shown).

We recently reported that potent Th1 responses to Ag85B-ESAT-6 may be elicited by the exquisite targeting/activation of a minute proportion of DCs following the use of another adjuvant formulation, IC31® [27], [40]. IC31® contains a KLK peptide and a non-CpG oligonucleotide which precipitate with antigen at the site of injection [28]. Following immunization, fewer than 3000 vaccine-associated cells per mice were retrieved from the draining LN, including 1500–2000 Adj^+^ (IC31®^+^) DCs and 500 Ag^+^Adj^+^ DCs. This is remarkably similar to the pattern elicited by Ag85B-ESAT-6/CAF01, which resulted into the same number of Ag^+^Adj^+^ LN DCs at 24 h after immunization (Fig. 4). We wondered whether this pattern resulted from limitations of lymphatic entry [45] or migration capacities of peripheral DCs [46]. However, immunizations with other lipid-based formulations resulted in 100-fold higher numbers of vaccine-associated LN DCs (manuscript in preparation). Thus, the exquisite targeting of a small number of DCs is an intrinsic property shared by both IC31® and CAF01, despite major biochemical differences. Another similarity between these two potent novel adjuvants is that they trigger DC recruitment, migration and activation during several days. Following injection of Ag85B-ESAT-6 in CAF01, both Ag^+^Adj^+^ and Ag^−^Adj^+^ DCs were recovered in the draining LN at higher numbers on Day 5 than on Day 1 (Fig. 5). Given the short half-life of activated DCs [47]–[49], this is likely to result from the sustained migration/activation of peripheral DCs from the injection site. The duration of this phenomenon is difficult to estimate: vaccine-associated cells were not retrieved from the draining LN on Day 21 after injection, which could be due to the detection limits of the method or the completion of Ag^+^/Adj^+^ DC migration. Indeed, a remarkable property of CAF01 is to elicit sustained CD4^+^ Th1 responses: the proportion of antigen-specific CD4^+^CD44^hi^ IFN-γ or TNF-α producing cells was similar 6 weeks and 6 months after immunization (Fig. 2). Adoptive transfer experiments are thus ongoing to address the hypothesis of prolonged DC recruitment at the injection site.

The CAF01 and IC31® formulations also rely on distinct immunomodulators: IC31® contains a non-CpG oligonucleotide that mediate its signaling through TLR-9 and IL-12 [28], whereas the TDB glycolipids of CAF01 activate the Syk-Card9-Bcl10-Malt1 signaling pathway [35]. A peculiarity of this pathway is that it does not only activate Th1 but also a strong Th17 response, which could play an important role in recruiting effector cells into the lungs at time of infection [8], [9]. This provided us with a unique opportunity to probe the function of the Syk-Card9-Bcl10-Malt1 signaling pathway in early life. The adult-like protection against a mycobacterial challenge mediated by CD4^+^ T cells, and the induction of adult-like neonatal Th1 and Th17 cells after 2 or even a single neonatal injection demonstrate that the activation of this pathway is indeed functional early in life (Table 1). Should TDB similarly effectively activate human neonatal DCs, in whom TLR-mediated signaling is often poorly effective [30], [31], this would identify the CAF01 formulation as a most promising adjuvant for vaccines against early life pathogens.

## Materials and Methods

### Mice

Specific pathogen-free C57BL/6 mice (7 days of age (1-week-old, neonates) or 8–12 weeks old (adult), Charles River) were bred and kept under specific pathogen-free conditions in the university zootechnology unit. Mice were immunized at the base of the neck using the subcutaneous (s.c.) route. For analyses of *in vivo* DC activation, the axial, brachial and auricular lymph nodes were harvested. Manipulations were conducted according to Swiss and European guidelines and experiments approved by the Geneva Veterinary Office.

### Antigen, Adjuvants and immunization

Recombinant Ag85B-ESAT-6 was prepared as described [50] and coupled with AlexaFluor 647 (Invitrogen, Basel, Switzerland). Dimethyldioctadecylammonium (DDA) bromide and a,a′-trehalose 6,6′-dibehenate (TDB) were purchased from Avanti Polar Lipids (Alabaster, AL) and the stable formulation designated CAF01 prepared by the lipid film hydration method as described [33]. To visualize CAF01, NBD-DPPE (1,2-Dipalmitoyl-sn-Glycero-3-Phosphoethanolamine-N-(7-nitro-2-1,3-benzoxadiazol-4-yl) : Avanti Polar Lipids, Alabaster, AL) was incorporated (4.7% w/w) during liposome preparation. Vaccines were formulated by absorbing antigens (5 or 15 µg) with CAF01 or Al(OH)_3_ (Alum, 1 mg, gift of Novartis Vaccines and Diagnostics, Siena, Italy). Buffer was used for control immunization. The addition of the fluorochrome did not affect the immunogenicity of the formulation (data not shown).

### Determination of T cell responses

Splenocytes were cultured with Ag85B-ESAT-6 (5 µg/ml) or medium alone. For determination of cytokine by ELISA or bioassay, supernatants collected after 72 h for quantification by ELISA of IFN-γ and IL-5 [20], TNF-α (BD Biosciences, San Diego, CA), IL-17 (R&D Systems, Abingdon, UK) and by bioassay IL-2 [51]. Under these conditions, blocking studies with antibodies against CD4 (clone GK1.5) and CD8 (clone H35-17.2) were undertaken. The antigen-specific IFN-γ-secreting T cells were quantified by ELISPOT, using Ag85B-ESAT-6 (2 µg/ml) or media alone for 48 h [52]. Cytokine expression (IFN-γ, TNF-α, IL2) and multi-functional T cell patterns following 6 hour antigen stimulation (5 µg/ml) of splenocytes were determined by intracellular staining (ICS), as previously described [27].

### Dendritic cell preparation, cell staining and flow cytometry

CD11c^+^ DC from draining LN were prepared by magnetic selection (Miltenyi Biotec, Bergisch-Gladbach, Germany) as described [27], [40]. Cells were pre-incubated with rat anti-CD16/32 mAb (2.4G2 clone), then stained with conjugated antibodies against CD11c (HL3 clone), CD11b (M1/70 clone), CD8α (53-6.7 clone), isotype controls (BD Pharmingen), CD86 (GL1 clone) (Biosource International, Camarillo CA), CD11c (N418 clone), CD40 (FGK45 clone), MHC class II (M5/114.15.2 clone), CD205 (NLDC-145 clone) (produced in house). Cells were further stained with streptavidin-PE or streptavidin-PECy7 (BD Pharmingen). Each sample was acquired on the FACSCalibur or FACSAria cytometers and data were analyzed using CellQuest Software (BD Biosciences) or FlowJo Software (Tree Star, Ashland, OR).

### Mycobacterial challenge

Six weeks after boost, mice were infected i.v with 10^7^ CFU of *Mycobacterium bovis* BCG Danish 1331. Four weeks p.i., mice were sacrificed and spleens and lungs homogenized for bacterial enumeration. Individual organs were plated in serial dilutions onto Middlebrook 7H11 agar and incubated for 3 weeks at 37°C prior to counting the number of CFU.

### Statistical analysis

Statistical analyses of the results obtained in various experimental groups were performed with the Mann-Whitney U test or ANOVA with Tukey test. Differences with probability values of >0.05 were considered insignificant.

## References

[1] Hoft DF (2008). Tuberculosis vaccine development: goals, immunological design, and evaluation.. Lancet.

[2] Baumann S, Nasser Eddine A, Kaufmann SH (2006). Progress in tuberculosis vaccine development.. Curr Opin Immunol.

[3] Kaufmann SH (2008). Rational design of novel antibacterial vaccines with an emphasis on tuberculosis.. Scand J Infect Dis.

[4] Giri PK (2008). How could we have better vaccines against tuberculosis?. Expert Opin Biol Ther.

[5] de Jong R, Altare F, Haagen IA, Elferink DG, Boer T (1998). Severe mycobacterial and Salmonella infections in interleukin-12 receptor-deficient patients.. Science.

[6] Reichenbach J, Rosenzweig S, Doffinger R, Dupuis S, Holland SM (2001). Mycobacterial diseases in primary immunodeficiencies.. Curr Opin Allergy Clin Immunol.

[7] Winthrop KL (2006). Risk and prevention of tuberculosis and other serious opportunistic infections associated with the inhibition of tumor necrosis factor.. Nat Clin Pract Rheumatol.

[8] Cooper AM, Khader SA (2008). The role of cytokines in the initiation, expansion, and control of cellular immunity to tuberculosis.. Immunol Rev.

[9] Khader SA, Bell GK, Pearl JE, Fountain JJ, Rangel-Moreno J (2007). IL-23 and IL-17 in the establishment of protective pulmonary CD4(+) T cell responses after vaccination and during Mycobacterium tuberculosis challenge.. Nat Immunol.

[10] Mandalakas AM, Starke JR (2005). Current concepts of childhood tuberculosis.. Semin Pediatr Infect Dis.

[11] Trunz BB, Fine P, Dye C (2006). Effect of BCG vaccination on childhood tuberculous meningitis and miliary tuberculosis worldwide: a meta-analysis and assessment of cost-effectiveness.. Lancet.

[12] Vekemans J, Amedei A, Ota MO, D'Elios MM, Goetghebuer T (2001). Neonatal bacillus Calmette-Guerin vaccination induces adult-like IFN-gamma production by CD4+ T lymphocytes.. Eur J Immunol.

[13] Miles DJ, van der Sande M, Crozier S, Ojuola O, Palmero MS (2008). Effects of antenatal and postnatal environments on CD4 T-cell responses to Mycobacterium bovis BCG in healthy infants in the Gambia.. Clin Vaccine Immunol.

[14] Soares AP, Scriba TJ, Joseph S, Harbacheuski R, Murray RA (2008). Bacillus Calmette-Guerin vaccination of human newborns induces T cells with complex cytokine and phenotypic profiles.. J Immunol.

[15] Scriba TJ, Kalsdorf B, Abrahams DA, Isaacs F, Hofmeister J (2008). Distinct, specific IL-17- and IL-22-producing CD4+ T cell subsets contribute to the human anti-mycobacterial immune response.. J Immunol.

[16] W.H.O. (2007). Global Advisory Committee on Vaccine Safety, 29–30 November 2006.. Wkly Epidemiol Rec.

[17] Siegrist CA (2001). Neonatal and early life vaccinology.. Vaccine.

[18] Mestas J, Hughes CC (2004). Of mice and not men: differences between mouse and human immunology.. J Immunol.

[19] Kovarik J, Martinez X, Pihlgren M, Bozzotti P, Tao MH (2000). Limitations of in vivo IL-12 supplementation strategies to induce Th1 early life responses to model viral and bacterial vaccine antigens.. Virology.

[20] Roduit C, Bozzotti P, Mielcarek N, Lambert PH, del Giudice G (2002). Immunogenicity and protective efficacy of neonatal vaccination against Bordetella pertussis in a murine model: evidence for early control of pertussis.. Infect Immun.

[21] Adkins B, Leclerc C, Marshall-Clarke S (2004). Neonatal adaptive immunity comes of age.. Nat Rev Immunol.

[22] Knuf M, Schmitt HJ, Wolter J, Schuerman L, Jacquet JM (2008). Neonatal vaccination with an acellular pertussis vaccine accelerates the acquisition of pertussis antibodies in infants.. J Pediatr.

[23] Dadaglio G, Sun CM, Lo-Man R, Siegrist CA, Leclerc C (2002). Efficient in vivo priming of specific cytotoxic T cell responses by neonatal dendritic cells.. J Immunol.

[24] Regner M, Martinez X, Belnoue E, Sun CM, Boisgerault F (2004). Partial activation of neonatal CD11c+ dendritic cells and induction of adult-like CD8+ cytotoxic T cell responses by synthetic microspheres.. J Immunol.

[25] Barrios C, Brandt C, Berney M, Lambert PH, Siegrist CA (1996). Partial correction of the TH2/TH1 imbalance in neonatal murine responses to vaccine antigens through selective adjuvant effects.. Eur J Immunol.

[26] Weeratna RD, Brazolot Millan CL, McCluskie MJ, Davis HL (2001). CpG ODN can re-direct the Th bias of established Th2 immune responses in adult and young mice.. FEMS Immunol Med Microbiol.

[27] Kamath AT, Rochat AF, Valenti MP, Agger EM, Lingnau K (2008). Adult-like anti-mycobacterial T cell and in vivo dendritic cell responses following neonatal immunization with Ag85B-ESAT-6 in the IC31 adjuvant.. PLoS ONE.

[28] Schellack C, Prinz K, Egyed A, Fritz JH, Wittmann B (2006). IC31, a novel adjuvant signaling via TLR9, induces potent cellular and humoral immune responses.. Vaccine.

[29] Riedl K, Riedl R, von Gabain A, Nagy E, Lingnau K (2008). The novel adjuvant IC31 strongly improves influenza vaccine-specific cellular and humoral immune responses in young adult and aged mice.. Vaccine.

[30] Levy O (2007). Innate immunity of the newborn: basic mechanisms and clinical correlates.. Nat Rev Immunol.

[31] Willems F, Vollstedt S, Suter M (2009). Phenotype and function of neonatal DC.. Eur J Immunol.

[32] Holten-Andersen L, Doherty TM, Korsholm KS, Andersen P (2004). Combination of the cationic surfactant dimethyl dioctadecyl ammonium bromide and synthetic mycobacterial cord factor as an efficient adjuvant for tuberculosis subunit vaccines.. Infect Immun.

[33] Davidsen J, Rosenkrands I, Christensen D, Vangala A, Kirby D (2005). Characterization of cationic liposomes based on dimethyldioctadecylammonium and synthetic cord factor from M. tuberculosis (trehalose 6,6′-dibehenate)-a novel adjuvant inducing both strong CMI and antibody responses.. Biochim Biophys Acta.

[34] Agger EM, Rosenkrands I, Hansen J, Brahimi K, Vandahl BS (2008). Cationic liposomes formulated with synthetic mycobacterial cordfactor (CAF01): a versatile adjuvant for vaccines with different immunological requirements.. PLoS ONE.

[35] Werninghaus K, Babiak A, Gross O, Holscher C, Dietrich H (2009). Adjuvanticity of a synthetic cord factor analogue for subunit Mycobacterium tuberculosis vaccination requires FcRgamma-Syk-Card9-dependent innate immune activation.. J Exp Med.

[36] Olsen AW, Williams A, Okkels LM, Hatch G, Andersen P (2004). Protective effect of a tuberculosis subunit vaccine based on a fusion of antigen 85B and ESAT-6 in the aerosol guinea pig model.. Infect Immun.

[37] Cendron D, Ingoure S, Martino A, Casetti R, Horand F (2007). A tuberculosis vaccine based on phosphoantigens and fusion proteins induces distinct gammadelta and alphabeta T cell responses in primates.. Eur J Immunol.

[38] Langermans JA, Doherty TM, Vervenne RA, van der Laan T, Lyashchenko K (2005). Protection of macaques against Mycobacterium tuberculosis infection by a subunit vaccine based on a fusion protein of antigen 85B and ESAT-6.. Vaccine.

[39] Korsholm KS, Agger EM, Foged C, Christensen D, Dietrich J (2007). The adjuvant mechanism of cationic dimethyldioctadecylammonium liposomes.. Immunology.

[40] Kamath AT, Valenti MP, Rochat AF, Agger EM, Lingnau K (2008). Protective anti-mycobacterial T cell responses through exquisite in vivo activation of vaccine-targeted dendritic cells.. Eur J Immunol.

[41] Henri S, Vremec D, Kamath A, Waithman J, Williams S (2001). The dendritic cell populations of mouse lymph nodes.. J Immunol.

[42] Siegrist CA (2007). The challenges of vaccine responses in early life: selected examples.. J Comp Pathol.

[43] Agger EM, Rosenkrands I, Olsen AW, Hatch G, Williams A (2006). Protective immunity to tuberculosis with Ag85B-ESAT-6 in a synthetic cationic adjuvant system IC31.. Vaccine.

[44] Tacken PJ, de Vries IJ, Torensma R, Figdor CG (2007). Dendritic-cell immunotherapy: from ex vivo loading to in vivo targeting.. Nat Rev Immunol.

[45] Teoh D, Johnson LA, Hanke T, McMichael AJ, Jackson DG (2009). Blocking development of a CD8+ T cell response by targeting lymphatic recruitment of APC.. J Immunol.

[46] Randolph GJ, Ochando J, Partida-Sanchez S (2008). Migration of dendritic cell subsets and their precursors.. Annu Rev Immunol.

[47] Ruedl C, Koebel P, Bachmann M, Hess M, Karjalainen K (2000). Anatomical origin of dendritic cells determines their life span in peripheral lymph nodes.. J Immunol.

[48] Kamath AT, Henri S, Battye F, Tough DF, Shortman K (2002). Developmental kinetics and lifespan of dendritic cells in mouse lymphoid organs.. Blood.

[49] Hou WS, Van Parijs L (2004). A Bcl-2-dependent molecular timer regulates the lifespan and immunogenicity of dendritic cells.. Nat Immunol.

[50] Weinrich Olsen A, van Pinxteren LA, Meng Okkels L, Birk Rasmussen P, Andersen P (2001). Protection of mice with a tuberculosis subunit vaccine based on a fusion protein of antigen 85b and esat-6.. Infect Immun.

[51] Gillis S, Ferm MM, Ou W, Smith KA (1978). T cell growth factor: parameters of production and a quantitative microassay for activity.. J Immunol.

[52] Martinez X, Regner M, Kovarik J, Zarei S, Hauser C (2003). CD4-independent protective cytotoxic T cells induced in early life by a non-replicative delivery system based on virus-like particles.. Virology.

